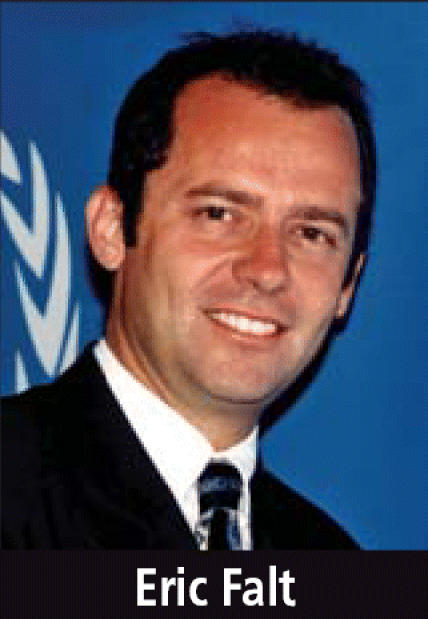# Guest Editorial: Sport and the Environment

**DOI:** 10.1289/ehp.114-a268

**Published:** 2006-05

**Authors:** Eric Falt

**Affiliations:** Division of Communications and Public Information, United Nations Environment Programme, Nairobi, Kenya, E-mail: eric.falt@unep.org

Pick up any newspaper or magazine these days and there will most likely be a prominent story about the environment and its relation to human health, well-being, or economic security. It seems that environmental sensitivity is moving from the fringe to center stage. No better confirmation can be found for this than the adoption of sustainability principles by major businesses. Bracketing those articles on the environment are full-page advertisements by energy companies and car manufacturers declaring their environmental credentials. “What’s good for the environment is good for the bottom line” is an increasingly common sentiment in the business community.

That quote could be attributable to any number of a new breed of environmentally conscious chief executive officers. In fact it is from Jack Groh, environment program director for the National Football League (NFL Enterprises 2006). The NFL is, of course, among United States’ most successful and high-profile corporate entities. Known both for its hard-nosed business acumen off the field, as well as the hard-hitting action on it, the NFL is one of a growing list of converts from the world of sport to a school of thought that incorporates environmental responsibility into the business model.

Increasingly, organizers of major environmental events are factoring the environment into their planning—and their publicity. According to the NFL (Anderson 2006), this year’s Super Bowl was carbon-neutral for the second year running. A tree planting campaign, in partnership with local groups such as the Boy Scouts, was designed to offset an anticipated 260 tons of carbon emissions generated by the event. Across the Atlantic, in the city of Torino, Italy, a similar initiative to reduce and offset carbon emissions featured as part of a raft of environmental components that formed an integral part of the XX Olympic Winter Games.

Teaming up with the world of sport has long been a priority for the United Nations Environment Programme (UNEP). Tasked by the UN General Assembly to “provide leadership and encourage partnership in caring for the environment by inspiring, informing and enabling nations and peoples to improve their quality of life without compromising that of future generations” (UNEP 2006), UNEP formed an alliance with the Olympic Movement back in 1994. As a member of the International Olympic Committee’s (IOC) Commission on Sport and the Environment, UNEP advises the IOC Executive Board on environment-related policy and, increasingly, works with bidding cities to refine the environmental component of their bids, monitor how well they have followed through on commitments, and help them raise environmental awareness during the events themselves (UNEP 2004).

The Torino Winter Olympics is probably the best example yet of a sport–environment collaboration that UNEP feels has significant promise in helping to further embed green principles throughout society. The organizing committee’s sustainability report (TOROC 2006b) demonstrates a detailed understanding of the environmental implications of staging a large-scale sporting event. It also shows the organizers’ commitment to integrating the principles of sustainability into all aspects of planning the Games. As well as the Heritage Climate Torino (HECTOR) project for making the games climate neutral (TOROC 2006a), the organizers implemented green procurement policies, reduced energy and water consumption, and monitored a wide range of environmental indicators, such as air quality and waste production.

It can be argued that such initiatives are like a drop in the ocean. However, there are two important points to consider. First, the axiom “think globally, act locally” is the kernel of environmental thinking. It is the opposite of throwing up your hands, saying “What’s the point?,” and waiting for others to take the lead. It is the spirit that led Rachel Carson to stand up to the combined power of government and industry. For many, *Silent Spring* (Carson 1962), which raised the alarm about the toxic effects of DDT and other pesticides, marked the dawn of an environmental movement that has not only seen the establishment of organizations such as UNEP and documents such as the 1992 Earth Summit’s Agenda 21 but also a growing coalition of seemingly unlikely allies, with environmental activists, hybrid car–driving celebrities, energy moguls, and evangelical Christians all lobbying the White House to face up to the challenge of climate change. Therefore, when organizations such as the NFL and the IOC say they are embracing environmental sustainability, their impact, however small, is never insignificant.

The second point is that sport has a vast environmental footprint. Consider just one example: athletic shoes. Most children and adults wear them. An initiative such as Nike’s “Reuse-A-Shoe” program (Nike 2004), which recycles old shoes to create new products such as basketball and tennis courts and athletics tracks, has to be better than adding to already overflowing landfills. This example of what we call “life-cycle thinking” is just one of a growing number of innovations that together could make a difference.

A difference is what we need. The reason the environment is front-page news is that things seem to be getting worse, not better. Glaciers are melting, hurricanes are getting fiercer, fisheries and other ecosystems are collapsing, and environmental degradation is driving the emergence or re-emergence of infectious diseases. UNEP’s job is to monitor these changes; alert our partners in government, business, and civil society to the dangers; and help them to identify effective responses. The world of sports is one such partner. It is a major industry in its own right, with considerable environmental impact. It is also a symbol and, we at UNEP believe, a powerful tool for advocacy.

Increasingly, we are finding sports personalities who are willing to speak out on behalf of the environment. They care because environmental change is affecting their sports. In the words of the Namibian sprinter Frankie Fredericks, “I breathe at least twice as deeply when I’m running. Air pollution is a threat to my health and my physical performance” (UNEP 2005). According to McConnell et al. (2002), in some communities in California where air quality is poor, the most athletic children are three times more likely to suffer from asthma than their peers who do not exercise.

Sports personalities also care about the environment because they are citizens of the world. Across the globe, children’s health is being damaged by environmental pollution. Millions die before their fifth birthday or have their intellectual and physical potential diminished by poor air quality, inadequate sanitation, and preventable diseases. The ultimate bottom line is the health and future of the world’s children. I believe that is why companies such as Nike and organizations such as the NFL and the IOC are increasingly recognizing the link between sport and the environment and are looking at ways of incorporating the principles of sustainability and environmental responsibility into what they do.

## Figures and Tables

**Figure:**